# Authors' Reply to Letter to the Editor: “Impact of Rehabilitation in the Neurointensive Care Unit on Long‐Term Survival in Patients With Traumatic Brain Injury”

**DOI:** 10.1111/aas.70353

**Published:** 2026-10-14

**Authors:** Kristin Alvsåker, Rolf Hanoa, Jon Michael Gran, Lisa Maria Högvall, Carl Johan Fredstedt Sogn, Theresa Olasveengen

**Affiliations:** ^1^ Postoperative and Intensive Care Department Oslo University Hospital (OUH) Oslo Norway; ^2^ Department of Physical Medicine and Rehabilitation Oslo University Hospital (OUH) Oslo Norway; ^3^ Department of Neurosurgery Oslo University Hospital (OUH) Oslo Norway; ^4^ Norwegian Institute of Basic Medical Sciences University of Oslo Oslo Norway; ^5^ Department of Anaesthesia and Intensive Care Oslo University Hospital (OUH) Oslo Norway; ^6^ Division of Medicine Institute of Clinical Medicine, University of Oslo Oslo Norway

## 1 Authors Reply

In their letter to the editor, Rosenkrantz et al. [1] highlight methodological features that may bias the observed effect in favor of early interdisciplinary rehabilitation (EIR) compared with standard care (SC) in our recent paper on the impact of neuro‐intensive care unit (NICU)–based rehabilitation on long‐term survival in patients with traumatic brain injury [2].

We thank the authors for their comments and agree that potential bias favoring the EIR group is an important concern that should be addressed in future studies. We clarify three main points raised by the authors.

### 1.1 Time Zero and Immortal Time Intervals

Rosenkrantz et al. are concerned about “different definitions of time zero between the two groups”. To elaborate what we write in the original paper, time zero for each subject was chosen with an aim for it to represent the initial day of eligibility for EIR. In our study, this corresponds to the stage at which patients have completed the neuro‐intensive care phase, sedation with short‐acting analgesics/sedatives has been discontinued, and weaning from organ support has begun. For patients in the EIR group, the start of follow‐up (time zero) was defined as the day EIR was initiated, typically as soon as sedation had been sufficiently tapered (median 12 days after injury). For patients in the SC group, the corresponding time zero was defined as the date of sedation discontinuation (median 6 days after injury), representing the same clinical stage at which EIR could in principle have been initiated.

Rosenkrantz et al. also argue that “EIR patients had to survive longer before the start of follow‐up” which “may have introduced immortal time intervals after the start of follow‐up”. However, by our design, there is no period in either group during which the outcome cannot occur [3]. Absence of observed deaths during the first week (which happen to be the case for both groups) is not a result of immortal time periods. Potential bias favoring the EIR group would therefore most likely be a result of conditional exchangeability and positivity violations [3].

### 1.2 Conditional Exchangeability and Positivity

Rozenkrantz et al. raise the argument that “EIR patients had longer NICU stays, a factor that again disproportionately favored the EIR group.” First of all, as described above, time zero in our study is not set at NICU discharge, but at the time of eligibility for EIR. The later timing of EIR initiation compared with SC (median 12 days for EIR vs. 6 days for SC) is not necessarily a source of bias. What is critical is whether the groups can be considered conditionally exchangeable at baseline after adjustment for baseline characteristics using inverse probability of treatment weighting. These baseline characteristics included the individuals' time since injury to start of follow‐up.

As noted in the original paper, time from injury to rehabilitation initiation varied according to injury severity, the need for neurosurgical intervention, treatment capacity, and referral pathways. The later median initiation of EIR and the longer NICU stay observed in the unadjusted cohort likely reflect that patients in the EIR group had more severe injuries and more frequently required neurosurgical procedures. In addition, the EIR intervention was delivered during the process of weaning from organ support within the NICU, whereas patients in the SC group were often weaned at local hospitals, resulting in shorter NICU stays for the SC group.

As in most observational studies, residual confounding, including confounding by indication, may persist. Educational level and other socioeconomic factors are known predictors of TBI long‐term outcomes [4, 5, 6]. Consequently, the possibility of unmeasured confounding should be acknowledged when interpreting the findings.

### 1.3 Target Trial Emulation

Rosenkrantz et al. conclude that “a target trial emulation may be a more general approach to compensate for delayed treatment initiation” and mention “a study with time‐varying exposure or a clone‐censor‐weight design” as alternative approaches [7]. They also argue that “the study would have benefited from further specifying when the randomization in a hypothetical RCT would have taken place”. We agree with the latter point, and hope that clarification provided here regarding our choice of time zero at which a hypothetical randomization of EIR and SC would take place addresses this issue.

A target trial emulation that allows for delayed treatment initiation could of course be considered. However, redefining time zero to the time of injury or admission would be to consider a different causal estimand and target population than in the target trial with a point treatment as in our study. Our hypothetical target trial can be described as a trial where patients eligible for EIR are randomized to either EIR or SC at the time they would receive EIR if it was available for everyone. Patients who die in the NICU and never would have been offered EIR would then, for example, not be eligible for such randomization. In fact, the inverse probability–weighted estimator used in our study can be viewed as a special case of a clone–censor–weight approach, tailored to a point treatment defined as initiation (or non‐initiation) of EIR at time zero.

## 2 Conclusion

Both residual confounding and statistical uncertainty may contribute to the observed hazard ratio of 0.56 in favor of the EIR group. The association between EIR and long‐term survival may also partly reflect downstream consequences at subsequent discharge destinations, potentially influencing clinical decision making related to survival. Despite these limitations, the results are encouraging and support continued evaluation of EIR in future studies designed to replicate and extend these findings.

## Author Contributions

Kristin Alvsaaker and Jon Michael Gran drafted the manuscript. Theresa Olasveengen supervised the manuscript. All authors revised and approved the final manuscript.

## Funding

The authors have nothing to report.

## Consent

The authors have nothing to report.

## Conflicts of Interest

The authors declare no conflicts of interest.

## Data Availability

Data sharing not applicable to this article as no datasets were generated or analysed during the current study.
